# Discussion on High and Low-Fusing Porcelains

**Published:** 1902-12-15

**Authors:** 


### DISCUSSION ON HIGH AND LOW-FUSING PORCELAINS.

BY THE ODONTOGRAPHIC SOCIETY OF CHICAGO.

As reported by the *Dental Review* for November, 1902.

Dr. A. E. Marey: The principal characteristics of the low-fusing bodies, are:

(1) A fusing point below that of pure gold.

(2) A large percentage of shrinkage.

(3) Low density and strength.

(4) Of a color which is limited in usefulness.

It must be admitted that a matrix of pure gold can be made a little more quickly and easily than a platinum one and it becomes less springy under the burnisher. Yet it is liable to be bent in handling and usually needs the support of investment, which takes time.

The shrinkage in low-fusing bodies is considerable —from about fifteen to thirty-three percent—and so this body must be worked with great care. The tendency to globulate in shrinkage necessitates careful additions to previous bakings. These bodies are very brittle and of low density and the danger of rendering them porous through overbaking is very great. Yet when properly worked they have sufficient strength to resist all ordinary mastication.

They ought never to be ground, as the highly glazed surface resists chemical disintegration and I do not think there is any excuse for the porosity caused by uncleanliness in working, or through overbaking. These I believe to be the principal causes for bubbling and crackling. Of course all porcelains are porous in proportion to the completeness of the fusing of their ingredients. The fusing *must then* be very sharply watched in the lower fusing bodies. They bake at a moderate heat and passing that temperature renders them entirely valuelesss.

There are some very desirable colors in this body. Yet when the fine, dense tooth substance in living teeth in the anterior part of the mouth is to be matched they are not so satisfactory for inlays as the higher-fusing bodies.

There is a use for these bodies of the better grade which I may properly call to the attention of those who have never so employed them, viz, over a biscuited surface of the higher-fusing bodies for large inlays, and this is applicable also to crown and bridge-work. A thin coating of this material, such as Ash's low-fusing body, well glazed over a high-fusing body biscuited, will make a very fine finish, uniting in a degree with the harder body it does not seem to be as fragile as when worked alone, and leaves a very dense body of porcelain beneath a fine overglaze which is usually so desirable.

Dr. J. S. Bridges: The word high-fusing simply implies that that particular porcelain fuses above the melting point of pure gold, about 1,800 degrees F. Any porcelain flowing below such a temperature has incorporated in its component parts a great portion of spar or similar ingredients, resulting in a more or less glassy mass, a condition of affairs our most expert makers of porcelain have striven for years to avoid, even since the earliest days, when a sort of Wedgewood ware was accepted as an apology for tooth restoration.

Porcelain operators strongly advocating the use of the low-fusing body for inlays base their plea principally upon the fact that they are enabled to utilize gold foil for a matrix, claiming it may be adapted to the walls of the most difficult and inaccessible cavities, where platinum foil would be prohibited because of its harshness and liability to split beyond all usefulness under manipulation.

Now, I unhesitatingly venture the assertion that platinum can be forced (not necessarily burnished) into the deepest, well-like cavities imaginable without a single break. But admitting such a contingency, it would be no detriment to the finished work, as either Brewster's or Close's porcelain bodies will bridge over an extensive tear.

With platinum accepted as a foundation for the porcelain body, I see no reason for hesitancy in choosing between the two classes offered, when we understand the qualities with which porcelain must be endowed to qualify for our consideration.

Ash, Whitely, S. S. White, Consolidated, Close and Brewster manufacture the principal high-fusing porcelains now on the market.

These porcelains range from one to twenty-five shades and are divided inlo foundation and enamel bodies, excepting Close, which is a one-color and one-body porcelain.

The foundation bisques in one to two minutes, and the enamel fuses to a complete gloss in one to one and a half minutes, after the melting point of pure gold.

Porcelain, to pass our requirements, should possess the greatest possible strength, both edge and en masse.

There should be hardness with a minimum degree of brittleness and no loss of density or tendency to become porous and grow spherical after repeated firings. After completion we should be enabled to grind and polish to a perfect surface if necessary.

The true color should be maintained throughout each and every baking. With a correctly laid color scheme our pigments should, after firing, result in an inlay that has but a small amount of translucency. Many of our most beautiful inlays become a hopeless spot of gloom after setting, because of the fading of the basal color, permitting the rays of light to pass entirely through the porcelain. The pigments employed in the higher-fusing bodies are less liable to do this than those in the lower-fusing.

Unless it is proven more conclusively than at present that the use of low-fusing bodies is more advantageous, we should be doing an injustice to ourselves and patients to employthem, for, except perhaps, a doubtful saving in time, I believe every proper characteristic attributed to the lower-fusing bodies is possessed in a greater degree by the high-fusing porcelains.

Robert Brewster: The requisites of dental porcelain may be summarized as follows:

*Strength*, that it may be applied with safety in any position in the mouth.

*Density*, that it may be ground and *absolutely polished* to present that smooth, dense, unabsorbent surface which experience has shown to be more congenial to the tissues than any other material.

*Stability of form.* It must possess the quality of retaining fine carved out lines under a high fire, or, as the potters call it, a "hard" fire.

*Color.* The shades of color to harmonize with natural teeth must be present in the porcelain, in addition to strength, density and stability of form. The color question is one of the most important with which the dentist has to deal. The beautiful results in inlay work which we frequently see, can only be produced with a porcelain which is so prepared that its shades are absolutely natural tints, and not primary colors. The only place for primary colors in porcelain work is where they can be used as paints, required occasionally to intensify, or modify, or to represent superficial stains or deposits.

Probably the most fascinating, and, at the same time educating portion of porcelain prosthesis will be found in the employment of high-fusing oil paints. The eye will become more discriminating and more readily able to detect natural from unnatural shades, the artistic sense of light and shade will become more pronounced.

The first step in the production of dental porcelain is the reduction of the refractory materials, silica, feldspar and kaolin (each with their own peculiar properties) to a condition to receive the metallic oxids or coloring agents. This reduction is made with alkalies, under heat. The extent to which the heat is carried depends upon the use to which the porcelain when made is to be subjected. If we want strength, that is, a high resistance under pressure and percussion, we must be careful to retain the elements of strength, and not have fluxed down our refractory and *very hard materials* below a certain point.

That point, experience and investigation have shown must not be below the melting point of pure gold, but somewhat higher. On the other hand, it must not be too high, or very undesirable dental porcelain will result.

For ascertaining exact degrees of heat, pyrometers are employed. In the higher branches of chemistry, as you are aware, electrical pyrometers are used. In the production of porcelain, metal pyrometers are usually employed, the standard of measure being pure gold. With this is alloyed platina in quantities varying from thirty percent to eighty percent, for the higher-fusing porcelains; with silver, for those fusing below the melting point of gold, and, for the easy-flowing enamels and glazes, silver and copper are alloyed together.

This universal practice of using a test of metal or other material as a standard by which to judge the degree of heat in a muffle or kiln is one that dentists can not afford to ignore if they wish to produce the best results; particularly so is this necessary when using the small rapid electric furnaces.

All porcelain has its own fusing point, that is to say, fuses at a degree of heat which developes its best qualities of strength, color and surface.

In the lower-fusing materials, those which may be fused with a blowpipe, for instance, it is possible to judge the heat with some degree of accuracy by simply observing the material melt, there not being sufficient brightness in the fusing material to cause any inconvenience to the eye. But with porcelain melting above 2,000° Fahr., in a small muffle, it would be very unwise to rely upon the eye alone to judge the correct time for withdrawing the heat, and for that reason I have always recommended the use of a cylinder of gold as a point of time in determining the heat of a muffle.

Large pieces of work, such as a full continuous gum denture, may be lifted out of the furnace and inspected, but small work, which loses its heat quickly by reason of its small bulk, should not be so treated. Porcelain should *never* be subjected to too sudden variations of heat, and the very best results, both for strength, color and translucency, are obtained by leaving the porcelain in the muffle to cool.

One of the disadvantages of low-fusing porcelain is its tendency under fire to become spherical. For this reason, and *and apart from its lack of the necessary amount of resistance under stress*, it does not appear suitable for extensive restorations or for general use in crown and bridge-work. The greatly increased amount of shrinkage which it exhibits, as compared with high-fusing porcelain, is not in itself of so much importance as its tendency to lose its form and destroy the artistic work which may have been expended upon it, and its further objectionable liability in some cases, to absorb secretions and change color.

The past has shown us that there has never been any difficulty in producing a material—having many desirable qualities—to fuse at about 1,600° Fahr., but the requisites for general porcelain work have never yet, in my experience, been found in a porcelain fusing below 2,200° Fahr. nor above 2,500°, taking the melting point of pure gold as 2010° Fahr.

For many years I have strongly advocated testing materials in the laboratory. It is some ten or twelve years since I made the first machine for testing the strength of porcelain teeth, and I hold that all materials intended for use in the mouth, where the question of *strength*, *solidity*, *expansion* or *contraction* is essential, should be submitted to rigid laboratory tests. Such tests, if scientifically conducted, supply the only *positive knowledge* obtainable.

Tests in the mouth are seldom reliable *in the matter of porcelain*, owing to the difference in bulk of material used—strength of the metal work supporting it—position in the mouth—nature of the occlusion—temperament of the patient, and so forth.

To ascertain for my own satisfaction the relative strength of the different porcelains, I made the following tests:

I selected a tooth mold used for making long pin plate teeth, and to ensure each test being identical, one space only in the mold was used, so that each tooth, when made, was exactly the same size in every direction. The platina pins were made from the same coil of wire, and in the same machine.

The teeth were then carefully dried and laid in the furnace and baked, each tooth being baked at its proper heat, which had previously been ascertained by pyrometer tests of the material in the same furnace.

The method of testing adopted in this case, is, I believe, very reliable, *as a comparative test.* My experience in testing various makes of teeth over several years is that each make has its own breaking point, and the average of each is maintained year after year.

It may be well to state here that some of the tests showed that porcelain which fused at about 2,200° Fahr. had greater strength than the regular porcelain teeth which fuse at about 2,780°. This was not the case, however, with porcelain which fused below 1,900° Fahr.

Dr. W. T. Reeves. In the use of porcelain I have had a limited experience in the use of low-fusing bodies. I have discarded them entirely for the reason that so much better results can be obtained from high-fusing bodies. You can obtain shades that will be reliable. You can manipulate your shades and vary them as you will from any shade at the neck of a tooth to a different shade at the cutting edge with high-fusing body. Different people vary in their way of handling porcelain to obtain results. In regard to my method of baking porcelain in layers, that can only be done successfully by the use of high-fusing body, and in connection with inlay work I think it is one of the means of doing away with what is called the shadow problem which is the bother of every inlay worker who attempts to mix a few bodies together and arrive at an approximate shade that you think will be the shade in the inlay, and then make the inlay all of that mixture. Starting in the matrix with a foundation layer and on that foundation lay the color desired, or lay in darker colors than are expected to be the ultimate outcome of the inlay, and over them bake another color that might be called an enamel color. This method produces the best result in inlay work. It certainly gives the translucent effect that you can get in no other way. With high-fusing body you can obtain a contour and hold it. You can finish your inlay so that it will be perfectly flush with the margin and not have any raised effect, or warted effect that you would get with the use of a low-fusing body. I think it is a mistake to make inlays with the expectation of grinding them after they are set. In a few places, like trimming the cutting edges of inlays in anterior teeth or the occlusal surfaces of inlays in molars and bicuspids, this could be done with a stone after setting, but those are the only places that I consider should be touched with a stone. If the necessity arises for altering the shape of your inlay it should be done previous to the stripping of the matrix and put in the furnace and glazed again. The use of primary colors in inlay work is something that will appeal to the artistic sense of everybody if they only start and try to use them. You have teeth that at the cutting edge have a steel-blue tinge, almost transparent teeth, and yet just the line above the cutting edge there is a grayish tinge. You can obtain that by painting a faint line of high-fusing body of a deep blue color and then overlaying that with other enamel body and allowing it to reflect through. The same way, if you have cusps of bicuspids or molars that have a brownish tinge under them, or gray, which often occurs, you can obtain that same effect with this high-fusing primary color and allowing it to reflect through what might properly be called the enamel color. In the use of high-fusing body in inlays you can obtain perfectly firm margins in which there is no danger of splintering; I doubt if this can be done with low-fusing body. The experience I have had with low-fusing body has been confined entirely to the low-fusing Downie body. I have yet to see any inlays made with the Jenkins body that compare with those that can be made with the high-fusing body.

DR. BENTLEY: Mr. Brewster said that porcelain that will fuse at 2,200° F. reaches its maximum usefulness. That is to say, the strength, density, stability of form and color seem to be gained at that point. Some bodies fuse at 2,700° and 2,800°, these are exceedingly high-fusing bodies. According to this statement there does not seem to be any advantage in having a body fuse at 2,800°. It adds nothing in strength, nothing in stability of form or color, nothing in density, but really detracts from these qualifications. He also states that low—fusing bodies, that will fuse below 1,814° also detract from these necessary qualifications. If I understand it correctly, Jenkin's body contains the same ingredients as the high—fusing body, with the addition of an alkali, sodium or potassium, to ruduce the fusing point. You can take any high—fusing body and add potassium or sodium to it and reduce the fusing point; but according to this test anything that is below the fusing point of gold does not preserve the essential elements of a proper inlay material. I do not admit that it is easier to burnish gold into a cavity than platinum. I believe that platinum can be burnished into a cavity with much more degree of certainty and fixity than gold, when you know how to do it, and it is not necessary to incorporate that platinum matrix into an investment as you would necessarily have to do with a gold matrix, which takes time. I think there is every advantage over the low-fusing body by using the high-fusing body.

DR. DON M. GALLIE: If there is any one important thing in connection with high or low-fusing body it is the question of color; to know how the color will last and how to obtain it. Mr. Brewster classified the different points that should go to make up a perfect body, and one of the strong points was that of color. Eight years ago I purchased a low-fusing body, and according to test this body was perfect. I became very enthusiastic on the subject. Everything that came along I reproduced in porcelain, and the result was that the chickens came home to roost one by one, and I found that the body was not serviceable. I recently began using the high-fusing bodies, and to my mind there is no comparison between the two. To discuss high and low-fusing body means to discuss the whole question of porcelain—the color, the edge strength, the matter of contouring and getting the results that we desire to get from the material. But many of us who are not as expert as Dr. Reeves will find that the greatest trouble we will have is in the color. You will find that different furnaces act in a different way. Dr. Bentley's furnace takes one minute longer to fuse than mine does. In my furnace I will fuse on one point two minutes, on the next point two minutes and the next one minute and get good results, and the next day by following the same course I will find that my porcelain has over-fused. I think the question to discuss also is how these bodies can be best used, and in the making of inlays one of the things to consider is the making of the matrix. Dr. Bentley says he does not think it is any easier to burnish a gold matrix into a cavity than a platinum matrix. I do. I can burnish it in easier, but I can't get it out. In reproducing one-third the cutting edge of a central incisor it is nice to have your platinum matrix to try over the tooth to see if the contour is right. For my part, I am very much in favor of high-fusing body. The greatest difficulty I have is in knowing just how to manipulate the different colors.

Dr. Hart J. Goslee. In the use and application of porcelain for our purposes there are four requirements that I would classify somewhat different from Mr. Brewster. The first requirement is that of strength; the second is that of stability of color; then stability of form, and lastly, translucency. An experience of eight years in the use of both high and low-fusing body, beginning with the low and ending with the high, has convinced me conclusively that we have practically no use whatever for any body that will fuse below 2,000° F., with the possible exception of some classes of extensive cavities where it is desirable to construct a porcelain restoration in the posterior part of the mouth. It may be indicated to some extent in such cases for the reason, I believe, that it is easier to burnish gold into a difficult cavity, and particularly if it is a large one, than to burnish a platinum matrix, and because color is not so important and strength is increased by the additional bulk. And I also believe that it is possible for me to prepare a cavity in a molar that Dr. Reeves or Dr. Bentley will admit can be better fitted with a pure gold matrix than with a platinum matrix by either burnishing or swaging. This point of ease and facility is the very reason why some have become so enthusiastic over low-fusing porcelain. They can adapt a gold matrix to a cavity with greater facility and greater ease. I predict, however, that the time will come when those who become expert and skillful will discard it and take up the high-fusing bodies, for the reason that they possess greater strength, that they hold their form and color better and that they are more translucent. I think the low-fusing bodies have a greater density than the high-fusing, and that the density of the body depends much upon its being properly fused. In the fusing of porcelain I am inclined to believe that no one will become exceedingly successful who depends altogether upon any test. I do not believe that anyone can properly fuse porcelain until he has learned to SEE WHEN it is fused. One reason why we depend upon tests is because a great many, and particularly beginners, are afraid to open the furnace and look at their work, in which there is no danger at all. The use of a test of pure gold is advantageous only in this respect, that you need not watch it at all up to the fusing point of the gold. From there on, however, you have to watch it just as carefully, until you get your exact time, at least, as you would have to do if you watched it all the way through. And I believe if you will endeavor to get along without the gold test that you will become so familiar with the heat and degree of incandescence that you can tell from the appearance of the furnace when it is necessary for you to begin to watch it closely. It is true and has been stated that in one furnace the body will fuse in two minutes after the fusing of the gold, and in another three minutes, and that variation exists with everybody and with every furnace. The voltage varies and is not always the same in the same furnace and the pressure of air likewise varies in the use of gasoline. To summarize with regard to fusion I would say that everyone who hopes to do successful porcelain work should not only familiarize himself with the character of the body, but also with the method of properly and accurately fusing it, and I predict that anybody who does become at all successful will learn to do it without the use of pure gold or any other test. If Mr. Brewster says that the maximum quality of strength in dental porcelains is to be obtained between 2,200° and 2,500°, then why not combine the possible advantages of both minimum and maximum, in one grade of body, and strike a happy medium? It has been said that the low-fusing body will give you a better enamel surface. This I am not prepared to believe, because the surface of porcelain, if it is at all fusible, is gained by carrying it up to the proper fusing point to obtain complete vitrification, and I get just as good an enamel surface on a body that fuses at 2,600° as one that fuses at 2,000°.

These fusing points are based upon the fusing point of pure gold as being 2,016° F.

DR. J. E. NYMAN: The point that Dr. Bentley dwelt upon, the point in Mr. Brewster's paper in regard to the fact that he had found that porcelain which fused at a certain temperature, 2,200°, was stronger than one that fused at 2,700°, should merely demonstrate one fact—i. e., that there is a proper relative proportion of the ingredients of that porcelain, and the moment you decrease the amount of flux and increase the amount of kaolin and the silicates, you raise the fusing point of that body and detract from its strength. Perhaps I can illustrate this better by referring to a material with which we are all familiar, and that is cement. There is a relative proportion of the powder and the liquid which will yield the best results. If you increase your amount of powder and decrease your amount of liquid you get a cement which is not as strong. The same is true in porcelain; if you make a porcelain body which has too much of any one ingredient in it you get a porcelain which does not fuse at the usual fusing point Mr. Brewster has referred to, and it does not therefore present the proper physical characteristics for our use. Now, I must take a little issue with Dr. Bentley's statement about the East having gone wild over low-fusing body, because it has only been within the last year that we have heard anything from the East on low-fusing porcelain. Many of the men down there have favored high-fusing body, and it was only after a visit of Dr. Jenkins that they began to advocate low-fusing body, and I do not know how much was due to the personal entertaining characteristics of Dr. Jenkins himself; I imagine that Dr. Jenkins has enthused these men with some of his own personality. Dr. Ottolengui made some statements in his paper which, to anyone who has had any experience in porcelain, were, to say the least, ridiculous. He stated that shrinkage in low-fusing body was less than in high-fusing body. That can be refuted in both clinical and laboratory tests. Another was that he could fit gold matrices into cavities where platinum matrices could not be fitted. You will note that I did not say that he could burnish gold into cavities that platinum could not be burnished into. I dispute that statement also. In his paper he also gave some illustrations of inlays, and if those were accurate illustrations could not get that matrix out of the tooth without springing it. Now there is another point which is an argument against low-fusing body, and that is that we are all variable quantities ourselves. There is a personal equation which should be taken into consideration in all our actions. If we should by any chance over-fuse a low-fusing porcelain five seconds longer than we should we might ruin that inlay, whereas if that was a high-fusing porcelain it would have no appreciable effect on it. Furthermore, the coloring matters that are used in tinting the low-fusing porcelains are not the same that are used in tinting the high-fusing. Most of the frits that are used for coloring high,fusing bodies are oxides, and you cannot get any tinting from these materials with a substance which fuses below that of gold. Therefore, they have had to cast around for some other metals to supply this color, and it just so happens that there is not a single color supplied in the low-fusing body which harmonizes in any degree whatever with the natural color of the teeth. So I cannot see any excuse whatever for using the low-fusing porcelain. In fact, there are many arguments against it. It lacks structural strength; it lacks harmony of color; there is danger of over-fusing it; there is danger, if you run it a little too long, of fusing your whole matrix, and there is just as much danger of tearing the matrix as there is with the platinum matrix, with the further disadvantage that you can not build low-fusing body over a tear in a gold matrix. So I can see no reason at all for parading the low-fusing body before the public.

Dr. Edmund Noyes: I would like to ask one question. The trend of this discussion seems to indicate that the exact fusing of porcelain is very important, that it should not be over-fused. Now is it possible to prepare for us platinum solders whose fusing point will correspond with the required fusing point of the porcelain with which they are made to use, so that instead of timing the furnace after the fusing of the pure gold, or watching it with the eye, we might have a platinum solder which would indicate, when it rolls into a ball, that it is just exactly at that point our porcelain is fused properly?

Mr. Brewster (closing the discussion): With regard to Dr. Goslee's estimate of the fusing point of the foundation body, he has probably arrived at that from a table giving the melting point of gold as above 2,000°, whereas my pyrometers are based on the melting point of gold being below 1,900°. No two authorities seem to agree as to the melting point of gold. In five authorities the range is from 1,886° to 2,200° F.

With regard to the particular use of the foundation body in inlay work, its value lies in rendering the matrix firm and unyielding prior to the application of the enamel body, the subsequent and repeated fusing of which does not affect the foundation body, and consequently causes no contraction of the matrix, a possible occurrence, if the whole mass fuses at each baking.

I see no good reasen for raising the fusing point of porcelain beyond what I have indicated, as I find by dynamometer tests that teeth made from the enamel body withstand a higher crushing strain than teeth which fuse at 2,800° F.

In reply to Dr. Noyes' question, metal tests such as he mentions are on the market, and may be obtained, but they are somewhat more expensive than pure gold.

